# Application of 3D Printing Technology for Design and Manufacturing of Customized Components for a Mechanical Stretching Bioreactor

**DOI:** 10.1155/2019/3957931

**Published:** 2019-04-21

**Authors:** Giovanni Putame, Mara Terzini, Dario Carbonaro, Giuseppe Pisani, Gianpaolo Serino, Franca Di Meglio, Clotilde Castaldo, Diana Massai

**Affiliations:** ^1^PolitoBIOMed Lab, Department of Mechanical and Aerospace Engineering, Politecnico di Torino, Turin 10129, Italy; ^2^Department of Public Health, University of Naples “Federico II”, Naples, Italy

## Abstract

Three-dimensional (3D) printing represents a key technology for rapid prototyping, allowing easy, rapid, and low-cost fabrication. In this work, 3D printing was applied for the in-house production of customized components of a mechanical stretching bioreactor with potential application for cardiac tissue engineering and mechanobiology studies. The culture chamber housing and the motor housing were developed as functional permanent parts, aimed at fixing the culture chamber position and at guaranteeing motor watertightness, respectively. Innovative sample holder prototypes were specifically designed and 3D-printed for holding thin and soft biological samples during cyclic stretch culture. The manufactured components were tested in-house and in a cell biology laboratory. Moreover, tensile tests and finite element analysis were performed to investigate the gripping performance of the sample holder prototypes. All the components showed suitable performances in terms of design, ease of use, and functionality. Based on 3D printing, the bioreactor optimization was completely performed in-house, from design to fabrication, enabling customization freedom, strict design-to-prototype timing, and cost and time effective testing, finally boosting the bioreactor development process.

## 1. Introduction

Tissue engineering aims to generate *in vitro* functional biomimetic substitutes to repair or replace damaged biological tissues and organs [1]. The three main components for *in vitro* tissue development are as follows: (1) cells, responsible for new tissue synthesis; (2) scaffolds, providing physical and structural support and biochemical cues for cells; and (3) biomimetic *in vitro* culture environment, for replicating the *in vivo* milieu and signals [2]. Numerous mechanobiology studies demonstrated that, in addition to cells, biomaterials, and chemical signals, physical stimuli play a fundamental role during the development of native tissues and for the generation of tissue engineered substitutes [3–7]. In this perspective, bioreactors are innovative and technological devices appositely engineered for providing *in vivo*-like culture conditions, by coping with limitations of conventional two-dimensional, static, and manual cell/tissue cultures. In particular, bioreactors are designed to culture cells/tissues in a three-dimensional (3D) environment, under monitored and controlled conditions (e.g., pH, O_2_, glucose, and lactate) and user-defined physical stimuli (e.g., stretching, compression, electrical pulse, and fluid shear stress). They can be used both as culture model systems, to investigate *in vitro* cell/tissue development and the effects of chemicophysical stimuli on tissue maturation, or as production systems, for finally directing stem cell fate and engineered tissue formation *in vitro* [8–14]. Moreover, when equipped with technological solutions for real-time closed-loop monitoring and control of culture conditions, bioreactors could allow for automated culture processes, leading to high quality, reproducible, and standardized cell/tissue cultures as required by industrial and clinical applications.

Since this is a relatively young and multifaceted research field, bioreactors are often high-cost customized devices designed and developed in-house, but usually prototypes and/or permanent parts are outsource manufactured by external companies. Indeed, they must satisfy strict design, quality, and functionality requirements imposed by the Good Laboratory Practices (GLP) adopted in cell biology/tissue engineering laboratories [15]. In particular, in such laboratories cell/tissue cultures are performed by using sterile equipment, working under laminar flow hood for sterility maintenance, and using incubators for temperature (37°C), humidity (85–95%), and carbon dioxide (5%) maintenance. Therefore, bioreactors should guarantee biocompatibility, sterilizability (preferentially by autoclave), ease of assembling and use, watertightness, and reliability for long-term culture processes in the incubator [16]. In this scenario, three-dimensional (3D) printing, based on computer-controlled layer-by-layer deposition of materials, represents a key technology both for rapid prototyping of components to be tested and for fabricating permanent functional parts. Indeed, unlike conventional subtractive machining processes (i.e., milling, turning, and drilling), 3D printing allows easy, rapid, and low-cost manufacturing of complex geometries by single-step processes. This entails clear advantages for the definition of the final bioreactor design and during the fabrication phase, in terms of design customization and flexibility, in-house manufacturing, and reduced times and costs for production and testing, leading to an overall improvement of the bioreactor development process. Recently, a small but growing number of groups are adopting 3D printing for the development of customized culture systems. Raveling and colleagues used fused deposition modelling (FDM) to manufacture a low-cost, highly customizable mechanical bioreactor for investigating soft tissue mechanics [17]. Schneidereit et al. 3D-printed a culture chamber with included electrodes for electrical stimulation and parallel microscopic evaluation [18]. Smith and colleagues developed a 3D-printed bioreactor platform designed for 3D-bioprinted tissue construct culture, perfusion, observation, and analysis [19].

In this work, we applied FDM-oriented design and manufacturing for the optimization of a recently developed mechanical stretching bioreactor with potential application for cardiac tissue engineering and mechanobiology studies, designed to provide cyclic uniaxial stretch to biological samples [20]. In particular, 3D printing was used to in-house fabricate the bioreactor culture chamber housing and the motor housing, functional parts designed to fix the culture chamber position and to guarantee motor watertightness, respectively. Moreover, innovative sample holder prototypes were specifically designed and 3D-printed for holding thin and soft biological samples and experimentally and computationally tested in terms of ease of use and gripping performance, before final production with biocompatible and autoclavable materials. Assembling, usability and functionality tests confirmed the excellent performances of all 3D-printed components, which strongly contributed to the bioreactor development process.

## 2. Materials and Methods

### 2.1. Bioreactor Setup

**Figure 1:**
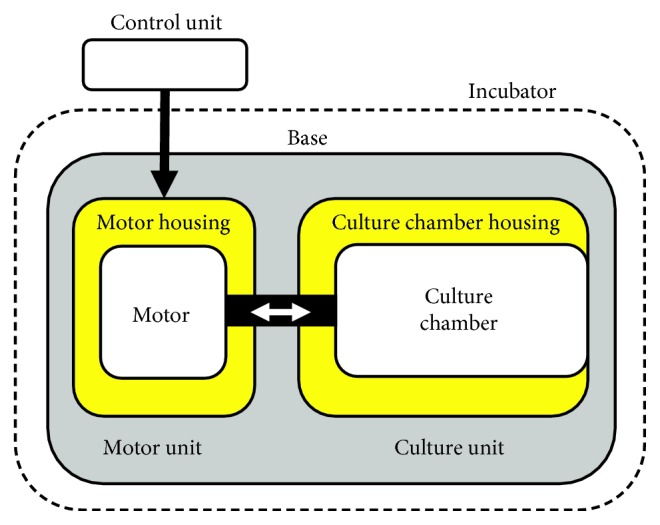
Schematic drawing of the bioreactor setup with the three modular subsystems: the culture unit, the motor unit, and the control unit. The functional FDM-printed components are represented in yellow. Both the motor housing and the culture chamber housing are bolted on a rigid planar base (represented in grey).

The bioreactor, which has been designed to be incubated and has been optimized with respect to a previous version [20], is composed of three modular subsystems: the culture unit, the motor unit and the control unit (Figure 1). The culture unit is dedicated to house the samples for sterile culture under mechanical stimulation. The motor unit, which has been modified and reduced in size, houses the motor, which is connected to the culture unit via a shaft for providing uniaxial mechanical stimulation. The control unit, whose development goes beyond the purpose of this study, enables the setting of the mechanical stimulation parameters and the motor control and must be located outside the incubator. In this setup, four bioreactor components (culture chamber housing, motor housing, and two sample holders) were designed following specific requirements and manufactured by FDM to be used either as permanent functional parts (culture chamber housing and motor housing) within the incubator or as working prototypes (sample holders) to test functionality and ease of use before to produce the final components in biocompatible and autoclavable materials.

### 2.2. FDM-Oriented Design and Manufacturing

#### 2.2.1. Design Requirements

The bioreactor design process was guided by the need to satisfy specific design requirements in terms of GLP-compliance, use, performance, and FDM manufacturing (Table 1). In particular, the whole bioreactor system should meet the GLP principles, which are rules and criteria for a quality system of organisational and working conditions in research laboratories to ensure consistency, reliability, reproducibility, and quality of nonclinical health studies [15]. Therefore, the bioreactor should be easy to assemble, to use, and to clean with the standard equipment (laminar hood, autoclave, incubator, gloves, and tweezers) and procedures of a cell biology/tissue engineering laboratory. With regard to dimensional requirements, the bioreactor must be placed on an incubator shelf (max 40 cm × 50 cm × 30 cm), and moreover, the new FDM-printed components must couple with preexisting parts (i.e., culture chamber, motor shaft, and connections). Due to the high humidity levels within the incubator, watertightness is mandatory in case of electrical and electronic parts for assuring reliable operation, especially for long-term culture processes. Modularity requirement comes from the need to guarantee accessibility and ease of use during assembling/disassembling and cleaning procedures, and together with customization, it allows providing adaptable, interchangeable, and scalable solutions for different cell/tissue applications to be used with the same bioreactor system. In particular, in this case, a specific design requirement for the gripping system came from the need to hold and culture under cyclic stretch thin and soft substrates/tissues for cardiac tissue engineering and mechanobiology studies. Finally, as regards the already existing culture chamber, where the components are in direct contact with the culture medium and/or with cells/tissues, the specific design requirements listed in Table 1 were previously fulfilled [20].

In parallel, because of the need to produce in-house customized components by a rapid, flexible, and low-cost process, FDM technology was selected for manufacturing. The FDM technology, developed by Stratasys Inc. (Eden Prairie, Minnesota, United States), is based on a filament of the thermoplastic material, and most commonly ABS [21], heated over its melting point, extruded through a nozzle and finally deposited to form structures composed of solidified layers [22]. Specific manufacturing design requirements are imposed by FDM technology, for which limitations exist in terms of minimum manufacturable size and features, such as holes and engraved details [23]. In detail, the thickness of supported and unsupported walls should be at least 0.66 mm. As regards the overhang inclination, for printability reasons, the maximum value is 45°; however, in this work, inclined surfaces and consequent stair-stepping effect were avoided for ease of cleaning needs. The minimum diameter for vertical holes is 2 mm, and the backlash between connecting parts is set equal to 0.1 mm. Lastly, threaded holes were avoided due to possible wear and tear caused by repeated assembling procedures.

#### 2.2.2. Design and Manufacturing

Taking into account the previously described design requirements, the culture chamber housing, the motor housing, and the sample holder prototypes were designed using the commercial computer-aided design (CAD) software Solidworks 2017 (Dassault Systemes, Vélizy-Villacoublay, France). In detail, the culture chamber housing was designed to fix the position of the removable culture chamber, to maintain a precise alignment and a defined distance between the culture unit and the motor unit and to fit the whole system within the incubator shelf. The motor housing was designed to be a watertight box protecting motor and electrical connections from incubator humidity. The sample holder prototypes, to be mounted within the culture chamber, were specifically designed to test the functionality of an innovative gripping system aimed at holding thin and soft biological samples [24, 25].

**Figure 2:**
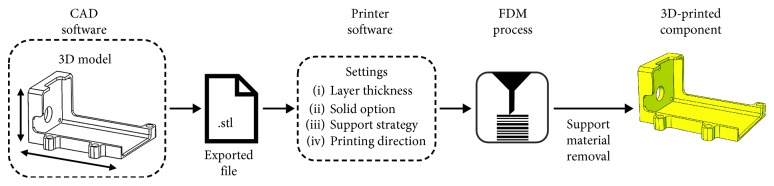
Design-to-manufacturing workflow.

The FDM manufacturing was performed uploading the design STL files on the printer software CatalystEX and using the 3D printer Stratasys uPrint SE Plus (Stratasys, Eden Prairie, Minnesota, United States). The ABS plus-P430 thermoplastic printing material (Stratasys, Eden Prairie, Minnesota, United States), durable enough to perform virtually the same as production parts (as declared by the manufacturer, physical properties listed in Table 2 [26]), was used in combination with the SR30 soluble support material. All components were printed setting the minimum layer thickness (0.254 mm) to minimize surface roughness and to optimize accuracy, imposing backlash (0.1 mm) to avoid interference during the mounting procedure and setting the solid fill option and the smart support strategy. For each component, the printing direction was defined balancing the resolution on circular features and the support material/time consumption, leading to different printing durations (the printer can build faster across the XY plane than it can along the *Z* axis) [27]. The support material was then removed manually, avoiding the use of the detergent bath.  Figure 2 shows the adopted design-to-manufacturing workflow. Printed components were then connected through nuts and bolts.

### 2.3. Testing

The 3D-printed components were tested in terms of assembly, coupling, and functionality of the whole bioreactor setup. Preliminary tests were performed in-house. In particular, the coupling between the culture chamber and the chamber housing was checked, and this latter was screwed on the rigid planar base. The motor housing was assembled and screwed on the rigid planar base. The sample holder prototypes were assembled and mounted within the culture chamber. The culture chamber mobile shaft was connected to the motor unit. Finally, the control unit was electrically connected to the motor unit, and an explanatory cyclic mechanical stimulation (displacement = 1 mm and frequency = 1 Hz) was run. In this phase, the following features were checked: coupling with preexisting components, holes alignment, culture unit, and motor unit alignment. Successively, trained laboratory operators tested the whole bioreactor system in a cell biology laboratory. In detail, the bioreactor culture chamber components were autoclaved, and the assembling procedure of the whole system was performed under laminar flow hood. To test the bioreactor functionality (including sterility maintenance), the culture chamber was filled with the culture medium, the assembled system was placed in an incubator, and the mechanical stimulation was switched on and run for 5 days. During the stimulation process, possible interferences between moving and fixed components were checked. At the end of the test, the watertightness of the motor unit, potential deformations of the 3D-printed components, and possible contamination were assessed.

Finally, the gripping performance of the sample holder prototypes was assessed performing both uniaxial tensile tests and finite element analysis (FEA) and compared with the performance of commercial titanium grips appositely designed for soft tissue characterization (TA Instruments, Inc, New Castle, DE, United States). For the experimental tests, one sample holder prototype was mounted on the material testing machine QTest10 (MTS Systems Corporation, Eden Prairie, Minnesota, United States) together with a contralateral commercial titanium grip. In order to assess the suitability of the prototype to hold thin and soft biological samples, two samples of human decellularized skin (length = 10 mm, width = 5 mm, and thickness = 0.6 mm) [28] were clamped between the sample holder and the titanium grip and then uniaxially stretched imposing a straining rate of 3.2% s^−1^ (gauge length = 5 mm) [29]. For comparison purposes, two additional samples were tested imposing the same conditions but using two titanium grips. Both loads and displacements were recorded to monitor the curve trend [30] because decreases in slope or sudden steps are indicators of gripping failure and slipping.

In addition, to investigate the contact pressure distributions exerted by the sample holder or the titanium grip configuration on the clamped samples, nonlinear FE analyses were performed using the software HyperMesh/HyperView 2017 (Altair Engineering, Troy, Michigan) and Abaqus 2017 (Dassault Systemes, Vélizy-Villacoublay, France). The sample holder and the titanium grip were modelled as rigid bodies, and only the surfaces in contact with the sample were discretized with the R3D4 elements. The sample was modelled as a rectangular parallelepiped (width = 5 mm and thickness = 0.6 mm), meshed with C3D8 elements, and considered as a linear-elastic material. To compare contact pressure results, the same sample contact surface (width = 5 mm and length = 3.2 mm) was modelled for both configurations. A frictionless contact between sample holder/grip surface and the sample was imposed. A force of 1 N was applied to the mobile part of the sample holder/grip, while the respective fixed parts were totally constrained.

## 3. Results and Discussion

### 3.1. FDM-Oriented Design and Manufacturing

**Figure 3:**
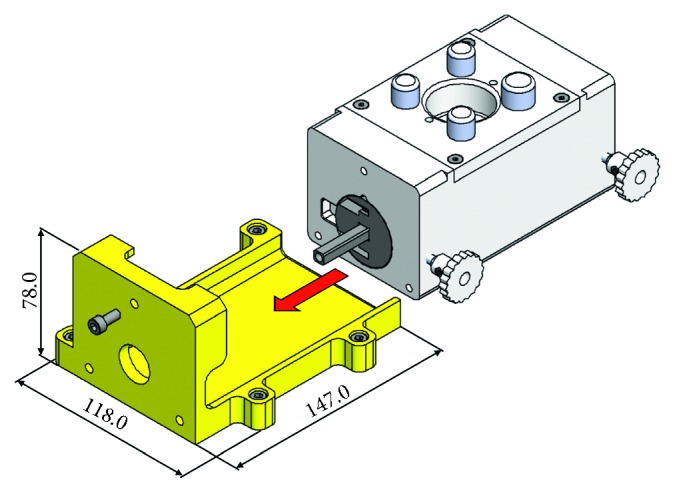
3D model of the culture chamber and its housing (in yellow). The red arrow shows the sliding direction for the coupling.

The culture chamber housing is a L-shaped chassis (width = 118 mm, length = 147 mm and height = 78 mm) composed of a planar base to house the culture chamber, two lateral edges, and a vertical wall designed to maintain the alignment and a defined distance between the culture unit and the motor unit (Figure 3). The coupling between the culture chamber and its housing is performed by sliding the culture chamber into the housing and fixing it through a screw (Figure 3).

**Figure 4:**
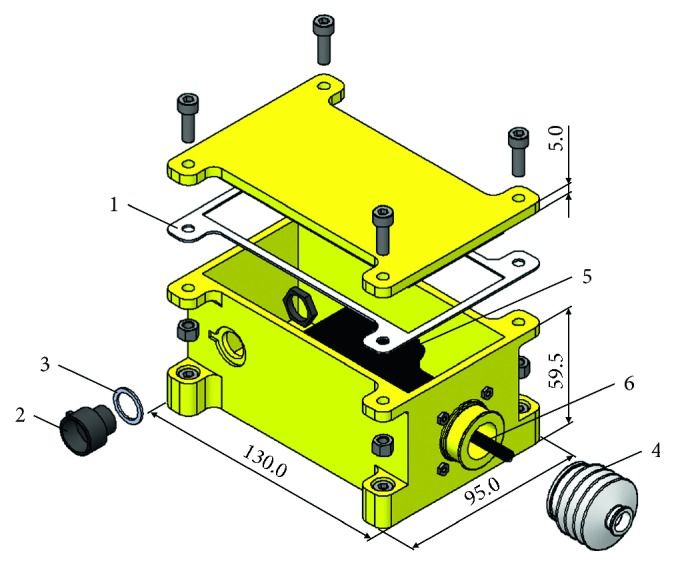
Exploded view of the 3D model of the motor unit. 1, silicon gasket; 2, waterproof socket; 3, gasket; 4, rubber bellow; 5, motor; 6, motor through shaft.

The motor housing is a watertight box (length = 130 mm, width = 95 mm, and height = 65.5 mm) designed for protecting the motor and electrical connections from incubator humidity and consists of two main parts: a container and a lid (Figure 4). To fix the lid on the container, four screw and nut sets are adopted. To guarantee watertightness, (i) a silicon gasket is placed between the container and the lid, (ii) a waterproof socket (including a gasket) for the connection with the control unit cable is screwed in a counterbore on the container lateral wall, and (iii) a rubber bellow for the motor through shaft fits on a protrusion of the container wall in front of the culture unit.

**Figure 5:**
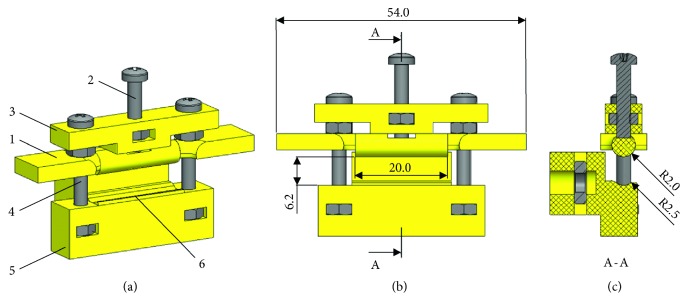
3D model of the sample holder prototype in open configuration. (a) Axonometric projection: 1, moving rod; 2, central screw; 3, planar guide; 4, lateral guide; 5, grip base; 6, hemicylindrical groove. (b) Front view. (c) Section view.

Guided by the design requirement of gripping thin and soft samples and inspired by commercial solutions for testing thin wires, the innovative design of the sample holder prototypes (maximum width = 54 mm, maximum height = 39.5 mm) is based on a guided moving cylindrical rod that, when pushed by a central screw connected to a planar guide, slides along two lateral guides against a grip base (Figure 5(a)). For gripping, the moving rod pushes the sample end against the grip base causing the sample wrapping around the cylindrical rod. To ensure a stable gripping of the sample, the proper coupling between the moving rod and the grip base is guaranteed by an appositely-sized hemicylindrical groove on the grip base (Figures 5(a), and 5(c)). The sample holder allows the gripping of samples with a maximum width of 20 mm. A maximum open position of 6.2 mm allows the positioning of the sample by using tweezers (Figure 5(b)). To fix the lateral guides on the grip base, dedicated cavities were designed to accommodate threaded nuts (Figure 5(b)).

**Figure 6:**
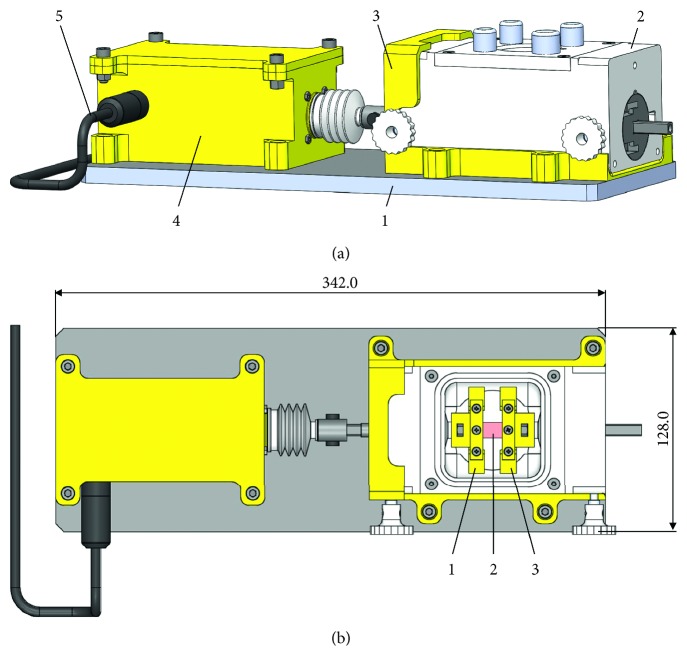
3D model of the whole bioreactor system. (a) Axonometric projection of the bioreactor with the following parts: 1, rigid planar base; 2, culture chamber; 3, culture chamber housing; 4, motor housing; 5, control unit cable. (b) Top view of the bioreactor 3D model without the culture chamber lid: 1, mobile sample holder; 2, sample; 3, fixed sample holder.

Figure 6 shows the assembled bioreactor system (with the exception of the control unit), composed of the culture unit and the motor unit screwed on the rigid planar base (length = 342 mm and width = 128 mm). The culture chamber is mounted on its housing and connected through a shaft to the motor placed inside the motor housing. The gripping system within the culture chamber consists of one mobile sample holder, screwed on the motor through-shaft, and a fixed sample holder screwed at the opposite culture chamber wall (Figure 6(b)).

The FDM manufacturing process required different durations depending on the printed component. For instance, notwithstanding their similar volumes, the printing process of the moving rod resulted more than four times longer than the process for manufacturing the planar guide, mainly due to the printing direction. Indeed, to guarantee the highest resolution on the rod surface, this component was printed with its long axis parallel to the *Z* printer axis. In Table 3 are listed printing times, printing material volumes, and support material volumes used for each component.

### 3.2. Testing

The in-house assembling of the 3D-printed components and their coupling with preexisting bioreactor parts did not show any issues. In detail, the position of the holes allowed screwing easily both the culture chamber housing and the motor housing on the rigid planar base. Similarly, the motor housing lid was easily coupled to the motor container. The culture chamber was slid into its housing, and the correct alignment with the motor unit was verified. The sample holder prototypes were assembled and mounted within the culture chamber. For all components, a suitable coupling with preexisting parts was confirmed.

**Figure 7:**
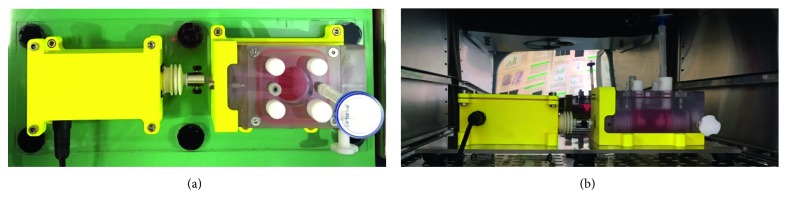
Assembled bioreactor: (a) top view of the bioreactor setup; (b) bioreactor within the incubator for the 5-day performance test.

Ease of use and performance of the whole bioreactor system were tested in a cell culture laboratory by trained laboratory operators. The assembling of the bioreactor components under laminar flow hood was easy and fast (Figure 7(a)). A performance test was carried out placing the bioreactor in incubator and running the mechanical stimulation for 5 days (Figure 7(b)). During working conditions, the system did not show any malfunction, no interferences between moving and fixed components were noticed, and the watertightness of the motor housing was confirmed. At the end of the test, neither deformations nor culture medium contamination was observed.

**Figure 8:**
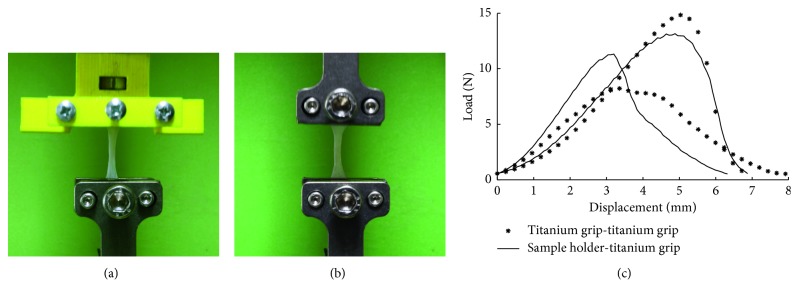
Gripping performance test of the sample holder prototype: setup of the testing machine QTest10 with the biological sample clamped by (a) the sample holder-titanium grip configuration and (b) the titanium grip-titanium grip configuration; (c) load-displacement curves obtained from the tensile tests performed with both configurations.

To test the gripping performance of the novel sample holder prototypes, uniaxial tensile tests of biological samples were performed using the testing machine QTest10 and two different gripping configurations: (i) sample holder–titanium grip (Figure 8(a)); (ii) titanium grip–titanium grip (Figure 8(b)). In the first configuration, each sample was easily positioned on the sample holder by using tweezers and firmly clamped by acting on the central screw, with the sample end wrapped around the moving rod. During the tests, no sample slipping was observed, and at rupture, each sample broke in the central cross section, confirming the proper load distribution in the gripping area. Similar results were obtained with the second configuration.  Figure 8(c) shows the four load-displacement curves, realigned at 0.5 N load, obtained from the tensile tests performed with both configurations. Besides the observable intravariability of mechanical behaviour typical of soft tissues, each curve increases smoothly up to rupture, with no sudden slope decrease, for both configurations. Tensile tests confirmed that the sample holder prototype is comparable to the commercial titanium grip in terms of gripping performance, and it is suitable in terms of FDM-based manufacturing.

**Figure 9:**
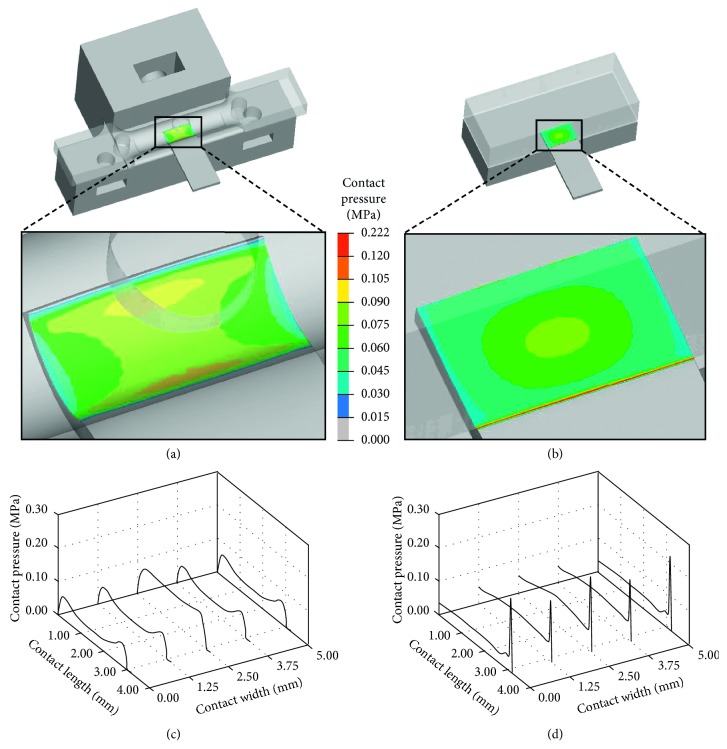
FEA results on contact pressure distributions on the sample surface exerted by (a, c) sample holder configuration and (b, d) titanium grip configuration.

In addition, FE analyses were performed to investigate the distributions of the contact pressure on the sample surface clamped by the sample holder or the titanium grip configurations. The FE results show that the sample holder configuration guarantees a more uniform contact pressure distribution on the sample surface (Figures 9(a) and 9(c); maximum contact pressure = 0.097 MPa) compared to the titanium grip, which causes a pressure concentration along the grip external edge (Figures 9(b) and 9(d); maximum contact pressure = 0.222 MPa). In case of thin and soft samples, contact pressure concentrations should be absolutely prevented since they could trigger sample break. The FEA results provided further confirmation of the suitability of the sample holder design.

With regard to possible limitations, components fabricated by FDM technology can be characterized by anisotropic behaviour, high surface roughness, poor geometry accuracy, and the presence of internal defects [31]. However, in this work, the manufactured components and the performed tests were not affected by such limitations.

## 4. Conclusions

In this work, 3D printing was applied for in-house designing and producing two functional parts and two prototype components for a mechanical stretching bioreactor with potential application for cardiac tissue engineering and mechanobiology studies. In detail, the 3D-printed culture chamber housing and motor housing met the design requirements of ease of use and functionality, guaranteeing alignment between the culture unit and the motor unit as well as the motor unit watertightness. The innovative sample holder prototypes, designed specifically for holding thin and soft biological samples, demonstrated their excellent performance in terms of ease of use and gripping, thus confirming the suitability of the design for future manufacturing in biocompatible and autoclavable materials. This approach enabled to perform in-house the entire bioreactor optimization process, from design to fabrication, providing customization freedom, strict design-to-prototype timing, and rapid and inexpensive testing. In conclusion, 3D printing technology allowed to manufacture low-cost, customized bioreactor components, and to reduce the risk of failure at later stages in new concept development, improving design and manufacturing process efficiency. In the next future, the increasing performances of printable materials in terms of biocompatibility, autoclavability, and transparency will further boost the development of low-cost bioreactors and customized culture devices. This will lead to more reproducible, standardized, and efficient basic studies, with great potential for the future routine production of tissue engineering strategies for clinical application.

## Figures and Tables

**Table 1:** Design requirements.

Design requirements
*Bioreactor system*
Ease of assembling, use, and cleaning
Small footprint (max 40 cm × 50 cm × 30 cm to fit in an incubator)
Watertightness (85–95% humidity in an incubator)
Modularity and customization
Reliability of culture processes (weeks in an incubator)

*Culture chamber components (in contact with the culture medium and/or cells/tissues)*
Cytocompatibility
Sterilizability (preferably by an autoclave)
Water/sterility tightness
Ease of assembly/disassembly under laminar flow hood
Ease of cleaning

*FDM manufacturing*
Supported and unsupported walls minimum thickness (0.66 mm)
Maximum overhang inclination (45°)
Minimum diameter of vertical holes (2 mm)
Backlash between connecting parts (0.1 mm)

**Table 2:** Physical properties of ABS plus-P430 [26].

*Mechanical properties* ^*∗*^	
Yield stress (MPa)	31.0
Ultimate stress (MPa)	33.0
Elastic modulus (MPa)	2.2
Elongation at break (%)	6
Elongation at yield (%)	2
*Thermal properties*	
Glass transition temperature (°C)	108

^*∗*^Uniaxial tensile tests performed along the printing direction of the specimens.

**Table 3:** Printing time, printing material volume, and support material volume for each 3D-printed component.

Component	Part	Printing time (h:min)	Printing material volume (cm^3^)	Support material volume (cm^3^)
Motor housing	Container	11:19	177.8	2.1
	Lid	2:29	83.5	7.8

Culture chamber housing		9:00	133.9	27.6

Sample holder	Moving rod	1:43	1.6	2.4
	Grip base	1:50	7.5	2.8
	Planar guide	0:23	1.8	0.6

## Data Availability

The data used to support the findings of this study are available from the corresponding author upon request.
